# CHCHD2 and CHCHD10: Future therapeutic targets in cognitive disorder and motor neuron disorder

**DOI:** 10.3389/fnins.2022.988265

**Published:** 2022-08-18

**Authors:** Tianlin Jiang, Yanli Wang, Xiaohong Wang, Jun Xu

**Affiliations:** ^1^Institute of Translational Medicine, Medical College, Yangzhou University, Yangzhou, China; ^2^Department of Neurology, Beijing Tiantan Hospital, Capital Medical University, Beijing, China; ^3^Jiangsu Key Laboratory of Experimental and Translational Non-coding RNA Research, Yangzhou University, Yangzhou, China; ^4^Jiangsu Co-innovation Center for Prevention and Control of Important Animal Infectious Diseases and Zoonoses, Yangzhou, China

**Keywords:** apoptosis, CHCHD2, CHCHD10, cognitive impairment, mitochondria, motor neuron injury

## Abstract

CHCHD2 and CHCHD10 are homolog mitochondrial proteins that play key roles in the neurological, cardiovascular, and reproductive systems. They are also involved in the mitochondrial metabolic process. Although previous research has concentrated on their functions within mitochondria, their functions within apoptosis, synaptic plasticity, cell migration as well as lipid metabolism remain to be concluded. The review highlights the different roles played by CHCHD2 and/or CHCHD10 binding to various target proteins (such as OPA-1, OMA-1, PINK, and TDP43) and reveals their non-negligible effects in cognitive impairments and motor neuron diseases. This review focuses on the functions of CHCHD2 and/or CHCHD10. This review reveals protective effects and mechanisms of CHCHD2 and CHCHD10 in neurodegenerative diseases characterized by cognitive and motor deficits, such as frontotemporal dementia (FTD), Lewy body dementia (LBD), Parkinson’s disease (PD) and amyotrophic lateral sclerosis (ALS). However, there are numerous specific mechanisms that have yet to be elucidated, and additional research into these mechanisms is required.

## Introduction

Coiled-coil-helix-coiled-coil-helix domain (CHCHD)-containing proteins are nuclear genes that encode mitochondrial protein (Liu et al., 2020a). The CHCH domain is characterized by a pair of cysteines separated by nine amino acids, knowns as the CX9C motifs (Liu et al., 2020a). The CHCHD-containing protein family is highly conserved in various physiological functions (Zhou et al., 2020).

CHCH mutation or deletion on cysteine residues will lead to a loss of mitochondrial functions associated with targeting and position. Apart from mitochondrial functions, the CHCHD family, especially CHCHD1, can also be found in the nucleus. Zhou et al. (2017) have summarized nine CHCHD-containing protein members and their major functions, implying roles in neurological disorders (Zhou et al., 2017).

CHCHD2 and CHCHD10 are imported to the mitochondrial intermembrane space and form a complex to regulate mitochondrial function (Martherus et al., 2010; Aras et al., 2013). CHCHD2 is involved in the regulation of oxidative phosphorylation as well as the inhibition of apoptosis (Modjtahedi et al., 2016). CHCHD10 is involved in regulating the activity of mitochondrial Cyclo-oxygenase (COX) and mitochondrial respiration during hypoxia (Ajroud-Driss et al., 2015). Many studies have shown that CHCHD2 and CHCHD10 are of great significance in mitochondrial dynamics, morphology, and functions (Funayama et al., 2015).

**FIGURE 1:**
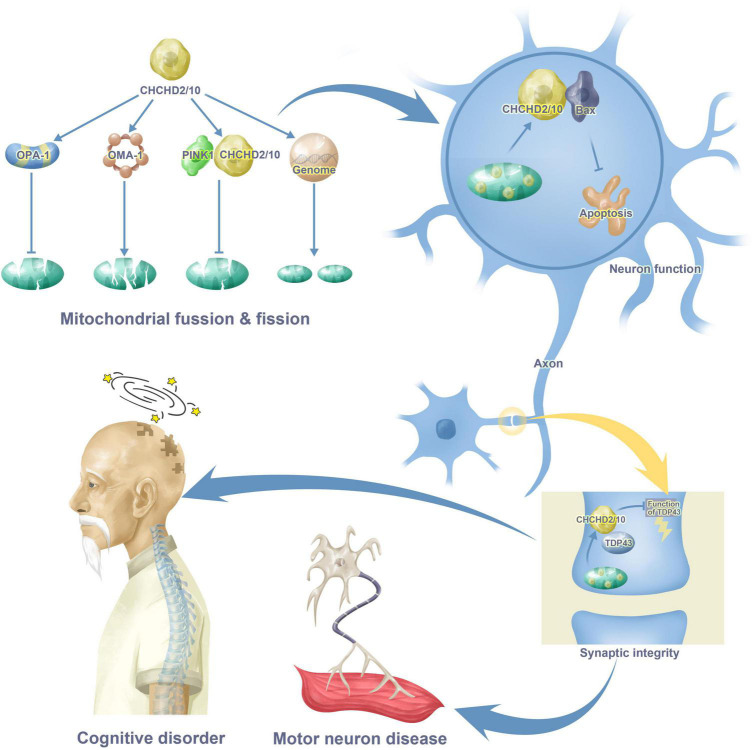
CHCHD2 and CHCHD10 functions in cognitive disorder and motor neuron disease. CHCHD2 and CHCHD10 play a role in mitochondria through mitochondrial fusion, fission and gene synthesis. CHCHD2 and CHCHD10 in mitochondria enter the cytoplasm of neurons through the combination with Bax, resulting in apoptosis. Moreover, CHCHD2 and CHCHD10 also appears in synaptic, binding to TDP43, inhibiting TDP43 neurotoxicity as a result. All these maladjustment contribute to cognitive disorder and motor neuron disease.

Increasing evidence has shown that CHCHD2 and CHCHD10 are associated with cognitive disorders and motor neuron diseases through their interactions with proteins such as OMA-1, OPA-1, TDP43, PINK, and p62. This review synthesizes evidence related to the functions of CHCHD2 and CHCHD10 and their molecular mechanisms in the progression of neurodegenerative diseases, providing implications for novel strategies in brain injury. Figure 1 provides a brief introduction to CHCHD2 and CHCHD10 functions in the nervous system.

## Roles of CHCHD2 and CHCHD10 in physiological and pathological processes

### CHCHD2 and CHCHD10 modulate mitochondrial dynamics, morphology, and respiratory chain

OPA1, OMA1, and DRP1 are mitochondrial proteins involved in fusion and fission processes. Among these, OPA-1 is necessary for mitochondrial fusion, and DRP1 is essential for mitochondrial fission (Mishra et al., 2010). In the absence of these dynamic processes, the accumulation of fragmented mitochondria may induce the clinical manifestation of mitochondrial-related diseases (Zhao et al., 2021). A previous study has found that CHCHD2 single knockout as well as CHCHD10 and CHCHD2 double knockouts are significantly reduced in mitochondrial fusion and fission (Ruan et al., 2022). It is important to highlight that changes in mitochondrial fusion and fission were unaffected by changes in cell viability after the knockdown of CHCHD2/10. In HELA cells with CHCHD2 single knockout, L-OPA1 decreases significantly and S-OPA1 increases under the action of OMA1 (Guo et al., 2020). Interestingly, a single knockout of CHCHD10 has no effect on the number of OPA-1, although a double knockout of CHCHD2/10 worsens the clearance of L-OPA1 (Liu et al., 2020b). DRP1, which promotes mitochondrial fission, is significantly reduced in CHCHD2 single knockout and CHCHD2/10 double knockout mice (Zhou et al., 2020). Thus, these findings suggest that the loss of CHCHD2/CHCHD10 may reduce mitochondrial dynamics. Other mitochondrial dynamics-related proteins, including MFN1, MFN2, and MFF, exhibit relatively similar levels of expression in control and CHCHD2/CHCHD10 knockdown cells (Zhou et al., 2020).

Cristae is the inner layer of the membrane inside the mitochondria and can be thought of as the basic bioenergy unit within the mitochondria (Nakamura et al., 2020). The multi-subunit complex present in the inner mitochondrial membrane (MICOS), which is concentrated at the cristae junction, regulates the maintenance of the cristae structure (Kondadi et al., 2020). CHCHD10 single knockdown cell lines had normal cristae structure, while CHCHD2 single knockdown cells, as well as CHCHD2 and CHCHD10 double knockdown cells, exhibit cristae abnormalities (Liu et al., 2020a,c).

The mitochondrial oxidative phosphorylation (OXPHOS) process can generate cellular energy in the form of ATP in eukaryotes (Garcia-Bartolome et al., 2020). A series of enzymatic complexes, including cytochrome c oxidase (COX, complex IV) are involved in this process. Guilt-by-association analysis (GBA) shows that CHCHD10 is likely involved in the regulation of COX activity, which is verified by vitro experiments (Liu et al., 2020a). CHCHD2, on the other hand, has been found to directly interact with COX to modulate COX activity, which influences mitochondrial membrane potential, ROS production, and cellular redox state (Zhou et al., 2020).

### CHCHD2 and CHCHD10 inhibit apoptosis

Apoptosis is essential for homeostasis (Chen, 2022). During apoptosis, the pro-apoptotic protein Bax activates and accumulates on the outer membrane of the mitochondria, increasing mitochondrial membrane permeabilization through an unknown mechanism.

Liu et al. (2015) discovers that CHCHD2 can inhibit mitochondrial apoptosis (Liu et al., 2015). CHCHD2 deletion can increase exposure to nuclear fragmentation and phosphatidylserine, both of which are apoptotic molecular markers. Besides, overexpression of CHCHD2 can reduce PARP lysis (Liu et al., 2015). CHCHD2 regulates Bax localization, activation, and oligomerization through interacting with Bcl-xL (Liu et al., 2015). Upon apoptotic stimulus, the level of CHCHD2 in the mitochondria decreases (Liu et al., 2015). A reduction in mitochondrial CHCHD2 levels is associated with a loss of Bcl-xL ability to inhibit Bax, allowing mitochondrial outer membrane permeabilization (MOMP), and apoptosis to continue (Liu et al., 2015). Overall, these findings imply that CHCHD2 acts as a negative regulator of apoptosis.

It is still unknown what roles CHCHD2 and CHCHD10 play in apoptosis. It is unclear how the interaction of CHCHD2 with Bcl-xl affects Bax activation. It is unspecific that indicators induced mitochondrial CHCHD2 depletion. The CHCH structural domain of CHCHD2 may act as a sensor for the redox changes that occur during mitochondrial apoptosis, resulting in appropriate CHCHD2 conformational changes that regulate Bax oligomers and MOMP. This hypothesis warrants further investigation. In addition, CHCHD2 may perform a nuclear role in regulating apoptosis at the mitochondrial membrane. These hypotheses require additional investigation.

### CHCHD2 and CHCHD10 modulate synaptic plasticity

Synaptic plasticity is the capacity of neurons to change the synapse in response to external activity over time (Noriega-Prieto et al., 2022). Since memories are thought to be programming of widely linked neuronal circuits, synaptic plasticity is one of the crucial neurochemicals, forming the basis of learning and memory (Grafe et al., 2022).

A study of Liu et al. (2022), via purified recombinant proteins, demonstrated that CHCHD10^*S*59*L*^ increased the size of TDP-43 aggregates but CHCHD10WT prevented TDP-43 aggregation. These changes paralleled the anomalies in motor neuron unit function, sciatic nerve action potential velocity, grip strength, and rotarod performance seen in CHCHD10^*R*15*L*^ and CHCHD10^*S*59*L*^ animals with functional deficits in long-term synaptic plasticity (Liu et al., 2022). They found that restoring CHCHD10WT in TDP-43 transgenic mice (TAR4; D10WT) decreased TDP-43 pathology and recovered TDP-43-induced deficits in long-term synaptic plasticity *in vivo* (Liu et al., 2022). Overall, these results indicate that CHCHD10-mediated modulation of TDP-43 aggregation in mitochondria is a substantial contributor to impairments in long-term synaptic plasticity and motor unit function.

In addition, roles of CHCHD2 and CHCHD10 in synaptic plasticity are unclear, while its role with TDP43 is recognized, its relationship with additional target proteins that can influence synapses requires further investigation.

### CHCHD2 and CHCHD10 promote cellular migration

Cell migration is an evolutionary conserved process which is essential for embryonic development, wound healing, immune response, angiogenesis, and cancer metastasis (Kurosaka and Kashina, 2008). Seo et al. (2010) have identified CHCHD2 as a gene that promotes cell migration and when CHCHD2 is overexpressed, it promotes cell migration, and when CHCHD2 is knocked down, it reduces cell migration. They concluded that CHCHD2-induced cell migration was associated with increased formation of actin stress fibers and adhesive patches, and CHCHD2 protein directly interacted with hyaluronan-binding protein 1, which possesses migration-inhibiting function, to balance cell migration (Seo et al., 2010). In addition, Wei et al. (2015) have shown that the copy number and protein levels of the CHCHD2 gene correlated with the epidermal growth factor receptor in non-small cell lung cancer in a co-amplified positive manner, and CHCHD2 is also thought to be an effector of cell proliferation, migration, and respiration, interacting with mitochondrial and extra-mitochondrial proteins in non-small cell lung cancer cell lines.

### CHCHD2 and CHCHD10 adjust lipid metabolism

In yeast, Mia40-regulated import is responsible for controlling the homeostatic state of mitochondrial phospholipids via Mia40/Erv1 import of the substrate Mdm35 carrying a CX9C2-type pattern (Modjtahedi et al., 2016). Mdm35 maintains stable interactions within the IMS with members of the evolutionary-conserved UPS/PRELI-like proteins UPS1 and UPS2 (Watanabe et al., 2015). These proteins are the mediators of the transfer of phospholipids between the outside and inner mitochondrial membranes. For example, UPS1 is responsible for regulating the movement of phosphatidic acid (PA) from the outer to the inner membrane (Modjtahedi et al., 2016). The reaction with a series of enzymes through PA eventually leads to the production of cardiolipin (CL).

## Cognitive disorder

### Frontotemporal dementia

Recent research has led to the discovery of a CHCHD10 genetic variation in patients with late-onset frontotemporal dementia (FTD) (Liu et al., 2020a). These individuals had mitochondrial myopathy and COX-negative fibers, both of which were related to numerous deletions in their mitochondrial DNA (mtDNA). In addition, aberrant crista structure was seen in patients’ fibroblasts, as well as decreased respiration activity and various deficiencies of respiratory chain components (Liu et al., 2020a). Therefore, they concluded that CHCHD10 mutations were the cause of FTD. Related protein-protein mechanisms are as below.

#### OMA-1

OMA1, an ATP-independent zinc ion metalloprotease expressed by the OMA1 gene, is also a redox-dependent protein with multiple transmembrane structural domains and zinc finger binding motifs located in the inner mitochondrial membrane (Hu et al., 2021). Mammalian OPA1 plays a role in a variety of cellular activities, including the construction of mitochondrial cristae, apoptosis inhibition, maintenance of mtDNA integrity, and oxidative phosphorylation, all of which interact with mitochondrial dynamics (Kaymak and Ryder, 2013).

**FIGURE 2:**
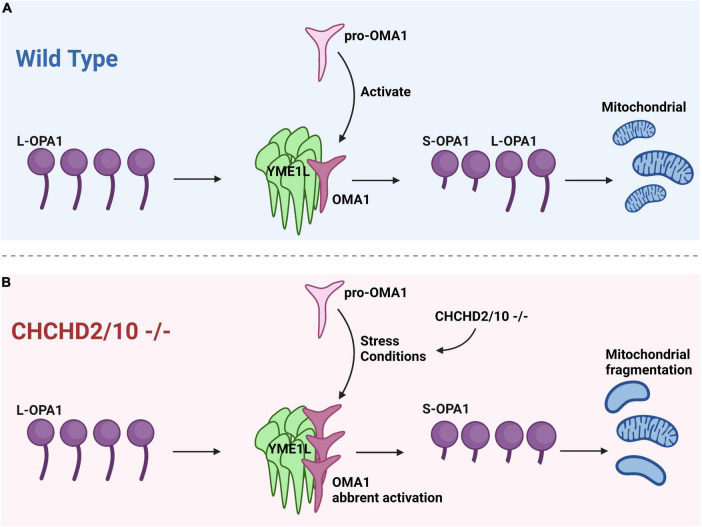
Cristae anomalies in CHCHD2/CHCHD10 linked to OPA1 processing by OMA1 activation. **(A)** Under normal conditions, when pro-OMA1 is activated, OMA1 binds to YME1L, resulting in the cleavage of L-OPA1 into S-OPA1 and the initiation of mitochondrial fission. Under normal conditions, L-OPA1 and S-OPA1 are in equilibrium, maintaining a balanced state of fission and fusion in mitochondria. **(B)** CHCHD2/10 deletion can result in excessive activation of pro-OMA1 and increased binding of OMA1 to YME1L, leading to mitochondrial fragmentation.

The OMA1-dependent OPA1 processing is affected by the loss of quality control proteases, such as YME1L, AFG3L2, and SPG7, as well as endosomal scaffolding proteins including SPFH family and SLP-2 (Liu et al., 2020c). OMA1 provides an escape route for cristae lacking protein quality control, separating defective mitochondrial units from the network for autophagy-mediated destruction (Liu et al., 2020a). It has been shown that CHCHD2/CHCHD10 double knockout may lead to cristae abnormalities due to increased stress-induced processing of L-OPA1 by the protease OMA1 in cell culture (Liu et al., 2020b; Figure 2).

Liu et al. (2020c) propose that mutant CHCHD2 or CHCHD10 may cause aberrant OMA1 activation via two possible mechanisms. One is that mutations in CHCHD2 or CHCHD10 may activate OMA1 at a lower concentration than wild-type (WT). If the concentration of CHCHD2/10 in cells reaches the threshold, OMA1 may be activated at physiological levels of CHCHD2 and CHCHD10 in the absence of aggregation. This mechanism is associated with the CHCHD10 G58R mutation, which causes myopathy and activates OMA1. Another is that CHCHD2 or CHCHD10 mutations may make proteins easily aggregate, resulting in an increase in total CHCHD2/CHCHD10 and a decrease in soluble CHCHD2/CHCHD10 (Liu et al., 2020c).

#### PINK1

Parkin is recruited by PINK1 to damage mitochondria in order to induce mitophagy (Chen et al., 2022). The PINK1 parkin-mediated mitophagy pathway has received a lot of attention (Quinn et al., 2020). Once PINK1 imports into the mitochondrial matrix, it will be cleaved by presenilin-associated rhomboid-like (PARL) protein, rendering it typically undetectable. Following being released into the cytoplasm, PINK1 fragments are destroyed by the ubiquitin-proteasome system through the N-end rule pathway. By directly interacting with parkin, cytosolic PINK1 fragments inhibit parkin translocation to mitochondria. PINK1 is stabilized in the OMM of injured, depolarized mitochondria, where it phosphorylates ubiquitin and parkin (at Ser65) to activate parkin’s E3 ligase activity and recruits Parkin from the cytosol to the mitochondria.

CHCHD10^*S*59*L*^ does not retain WT -like activity, indicating a dominant-negative mechanism. CHCHD10^*S*59*L*^ expression caused mitochondrial PINK1 stability, and genetic/pharmacological suppression of PINK1 alleviated CHCHD10^*S*59*L*^-induced cell toxicity (Baek et al., 2021). According to previous studies, decreasing PINK1 or parkin-mediated pathways is beneficial *in vivo* illness models of SOD1, FUS, and TARDBP mutations, according to prior research (Baek et al., 2021).

### Lewy body dementia

Clinically, Lewy body dementia (LBD) is the second most common neurodegenerative dementia after Alzheimer’s disease (AD), with clinical manifestations of fluctuating cognitive impairment, Parkinson’s syndrome, and psychiatric symptoms highlighted by visual hallucinations (Berman and Miller-Patterson, 2019). Ogaki et al. (2015) collected 610 patients with pathologically confirmed DLB and these patients underwent whole-exome sequencing of CHCHD2 gene and nine rare but not clinically significant exon mutations were detected: p.P2L, p.G4R, p.P14S, p.A16A, p.V31V, p.P34L, p.A37V, p.A49V, and p.A93V, eight of which were located in within the mitochondrial targeting sequence (MTS) of the CHCHD2 gene. In spite of the role of CHCHD2 gene mutations in DLB remains for further clarification, the possibility that rare variants in the mitochondrial targeting sequence are risk factors for LBD cannot be ruled out (Kee et al., 2022).

## Motor neuron disorder

### Amyotrophic lateral sclerosis

CHCHD10 mutations associated with FTD/amyotrophic lateral sclerosis (ALS) have been shown that CHCHD 10 mutations impair ability to bind both OPA1 and mitofilin, and these mutants reduced the molecular weight of endogenous CHCHD10, mitofilin, and OPA1 (Zhou et al., 2017). And these mutations are associated with mitochondrial fusion and respiration (Xiao et al., 2020). In the brains of human FTLD-TDP and TDP-43 transgenic mice, TDP-43 causes a reduction in CHCHD10 while also disrupting the natural complexes of CHCHD10, OPA1, and mitofilin (Penttila et al., 2017). TDP-43 is responsible for the mitochondrial defects linked with CHCHD10 mutations, although wild-type CHCHD10 is able to rescan (Liu et al., 2020a). Detailed mechanisms are as below.

#### OPA-1

OPA1 is heavily regulated by mitochondrial bioenergetics and proteostatic stress to dynamically shape the inner membrane in response to the changes in the mitochondrial network (Khin et al., 2021). When mitochondrial stressors activate the peptidase OMA1, it cleaves the active long-form of OPA1 (L-OPA1) from its membrane anchor, resulting in mitochondrial fragmentation and alterations in cristae structure (Morio et al., 2021).

**FIGURE 3:**
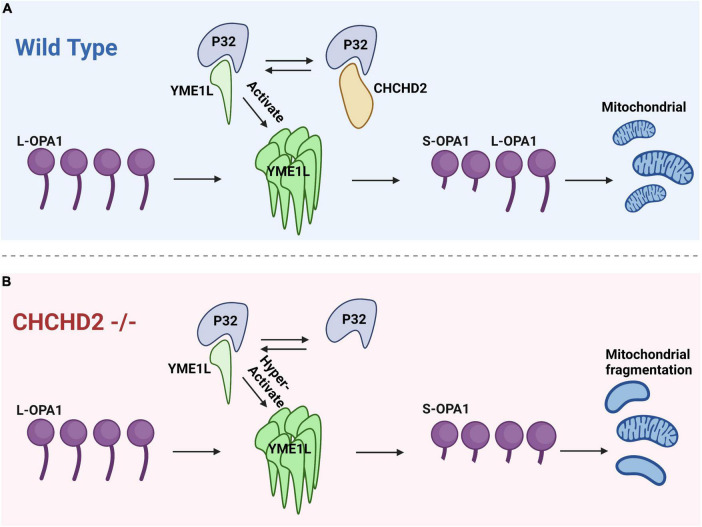
The interaction between CHCHD2 and P32 regulates the levels of OPA1 to modulate mitochondrial fission. **(A)** In the wild type groups, P32 binds to CHCHD2 and YME1L. The interaction between P32 and YME1L enhances OPA1 degradation. CHCHD2 competes with YME1L for P32, and as a result, OPA1 levels are maintained. **(B)** In CHCHD2 mutants, YME1L has access to a greater quantity of P32, which facilitates the degradation of OPA1 and leads to mitochondrial fragmentation.

Liu et al. (2020b) have found that YME1L expression in flies reduces Opa1 levels. p32, a binding partner of CHCHD2, also binds to YME1L to increase YME1L activity, so CHCHD2 competes with YME1L to interact with P32 and decrease YME1L activity (Liu et al., 2020b). It is surprising that fly YME1L could reduce Opa1 levels because the mammalian homolog of YME1L is required for OPA1 fusion activity (Liu et al., 2020a). The L-OPA1 form is necessary for mitochondrial inner membrane fusion, whereas the S-OPA1 form promotes inner membrane fission. L-OPA1 and S-OPA1 participate together in balancing the mitochondrial fusion-fission process. It has been previously proposed that CHCHD2 regulates Opa1 levels by competing with YME1L for P32 binding, thereby regulating mitochondrial fusion; even though YME1L activity increases upon binding to P32, deletion of CHCHD2 increases YME1L binding to P32, increases YME1L activity, and decreases L-Opa1 levels, resulting in mitochondrial fission (Liu et al., 2020b). As Figure 3 depicts, decreased binding of the CHCHD2 mutant to P32 leads to a corresponding increase in YME1L activity, addressing some of the deficiencies of the CHCHD2 mutant (Liu et al., 2020b). In this study, they have found that disease-associated variants of CHCHD2 proteins retain their ability to bind to P32. More studies are needed to determine how these mutant variants of CHCHD2 affect CHCHD2 function.

#### TDP-43

TAR DNA-binding protein 43 (TDP-43) is a heterogeneous ribonucleoprotein (hnRNP) nuclear protein that regulates RNA splicing, stability, and transport (Candia et al., 2022). TDP-43 is frequently detected in the cytoplasm of abnormal neurons, where it is ubiquitinated and fragmented, making it prone to aggregation (Smirnov et al., 2022). Although TDP-43 mutations are detected in a small fraction of ALS and FTLD-TDP cases, TDP-43 pathology is linked to the overwhelming majority of ALS and FTLD cases (Kee et al., 2021). Increasing data suggest that TDP-43 appears to be particularly neurotoxic, in large part because it induces mitochondrial malfunction (Smirnov et al., 2022). TDP-43 is associated with mitochondrial co-localization, promoting mitochondrial fission and leading to abnormal mitochondrial transport.

The malfunction of CHCHD10 results in cytoplasmic TDP-43 accumulation (Woo et al., 2017). CHCHD10 formed physical complexes with TDP-43, which required intact N-terminal 16 residues, and TDP-43 boosted the nuclear localization of CHCHD10 through retrograde signaling, which was linked with an increase in nuclear-encoded and mitochondrial-targeted transcripts (Zhou et al., 2017). In contrast to WT CHCHD10, FTD/ALS-associated CHCHD10 mutations (R15L and S59L) cause TDP-43 cytoplasmic mislocalization in irregularly shaped inclusions that often co-localized with mitochondria (Woo et al., 2017).

### Parkinson’s disease

Parkinson’s disease (PD) is characterized by degeneration of dopaminergic neurons in the dense nigrostriatal region and the formation of Lewy bodies, which usually manifest clinically as resting tremor, myotonia, and bradykine (Corti et al., 2011). CHCHD2 mutations are associated with both late-onset autosomal dominant PD and sporadic PD (Zhou et al., 2017). To date, three CHCHD2 genetic mutants have been identified in autosomal dominant PD patients: c.182C > T (p.Thr61Ile), c.434G > A (p.Arg145Gln) and c.300 + 5G > A. Funayama et al. (2015) identified by second generation sequencing in a Japanese PD family line. The c.182C > T (p.Thr61Ile) heterozygous mutation in the CHCHD2 gene was discovered by second generation sequencing in a Japanese PD family, and it was found in eight PD patients from two generations of the family. Next, Funayama et al. (2015) expanded their sample and identified two other mutations in CHCHD2 that may be associated with PD in 340 patients with familial PD in Japan: c.434G > A (p.Arg145Gln) and c.300 + 5G > A; and a single and leotide polymorphism in the CHCHD2 gene was found in 517 patients with sporadic PD c.- 9T > G (rs10043) and Pro2Leu (c.5C > T; rs142444896), which increased the risk of developing sporadic PD in the Japanese population by 2.51-fold and 4.96-fold, respectively. In a recent study, four familial PD patients from Western Europe, p.Ala32Thr, p.Pro34Leu, and p.Ile80Val mutations in the CHCHD2 gene were also seem to be potentially associated with familial PD (Foo et al., 2015). Furthermore, Meng et al. (2017) discovered that deletion of CHCHD2 in *Drosophila* leads to abnormalities in its gene structure and impaired mitochondrial oxygen respiration, resulting in PD pathological mechanisms such as oxidative stress, loss of dopaminergic neurons, and motor dysfunction. These findings support the hypothesis that mutations in CHCHD2 may cause mitochondrial dysfunction and participate in the pathological process of PD.

Table 1 shows the CHCHD2 and CHCHD10-related mechanisms in the diseases addressed above.

**TABLE 1:** Current findings in CHCHD2 and CHCHD10 involved mechanisms in diseases.

CHCHD2 change	CHCHD10 change	Mechanisms	Overall impact	Disease(s)	Reference
knockdown	–	Bax binding ↓; Bcl-2 binding ↓	Apoptosis ↑	Cancers (prostate, lung, kidney, skin, brain, mesothelium head and neck); Leukemias (Acute myeloid and childhood T-cell acute lymphoblastic)	Liu et al., 2015
knockdown	–	C1QBP binding ↓; YBX1 binding ↓	Cell proliferation ↓Cell Migration ↓; Mitochondrial respiration ↓	Non-small cell lung carcinoma	Wei et al., 2015
knockdown	–	L-OPA-1 ↓; S-OPA-1 ↑	Mitochondrial fragmentation ↑	ALS	Liu et al., 2020b
–	knockdown	SLP2 binding ↓	Prohibitin stability ↓	ALS; FTD	Genin et al., 2022
knockdown	knockdown	OMA-1 activation↓	Mitochondrial fragmentation ↑	ALS; FTD; myopathy	Liu et al., 2020c
–	CHCHD10^S59L^	PINK1 phosphorylation ↑	Mitochondrial fragmentation ↑; Cell toxicity ↑	ALS; FTD	Baek et al., 2021
–	CHCHD10^R5L^; CHCHD10^S59L^	cytoplasmic TDP-43 accumulation ↑	Mitochondrial-targeted transcripts ↓; Neurotoxic ↑	ALS	Chia et al., 2018

## Conclusion

CHCHD2 and CHCHD10, as mitochondrial inner membrane proteins, regulate apoptosis, synaptic plasticity, cell migration, and lipid metabolism, in addition to their essential activities in mitochondria. Furthermore, the ability of these proteins to carry out their functions is dependent on their interactions with a wide number of other proteins, including OPA1, OMA1, Bcl-xL, Bax, PINK, and TDP43. In the long run, they can regulate neurological disorders such as LBD, FTD, PD, ALS, and other neurological diseases through these responses. Furthermore, the research that has been done on the effects of CHCHD10 on the reproductive system has seen some progress, while more efforts in this field worth to be taken.

However, the majority of the recent studies have remained at the level of genetic analysis. Their mechanisms on these diseases still require more investigations and additional signaling pathways should be investigated. It is possible to examine the mechanism of action of CHCHD2 and CHCHD10 on neurological illnesses by using developing bioinformatics methods. The results of these investigations can then be corroborated with wet experiments in order to be corroborated. If the therapeutic effects of CHCHD2 and CHCHD10 are further confirmed, it will be necessary to conduct additional and more in-depth experimental studies to determine how they can be applied to the clinic.

## Author contributions

JX designed the review. XW revised the article and made language touch-ups. YW made changes to the figures and made grammatical corrections. TJ completed the writing of the review, the drawing of the figures, and the production of the table. All authors contributed to the article and approved the submitted version.
